# Linking stream ecology with morphological variability in a native freshwater fish from semi-arid Australia

**DOI:** 10.1002/ece3.1590

**Published:** 2015-07-16

**Authors:** Samantha Lostrom, Jonathan P Evans, Pauline F Grierson, Shaun P Collin, Peter M Davies, Jennifer L Kelley

**Affiliations:** 1 School of Animal Biology (M092), The University of Western Australia35 Stirling Highway, Crawley, Western Australia, 6009, Australia; 2 Ecosystems Research Group and West Australian Biogeochemistry Centre, School of Plant Biology, The University of Western Australia35 Stirling Highway, Crawley, Western Australia, 6009, Australia; 3 UWA Oceans Institute (M470), The University of Western Australia35 Stirling Highway, Crawley, Western Australia, 6009, Australia; 4 Centre of Excellence in Natural Resource Management, The University of Western AustraliaAlbany, Western Australia, Australia

**Keywords:** Local adaptation, phenotype–environment correlation, phenotypic plasticity, Pilbara, polymorphism, population differentiation

## Abstract

Environmental variation is a potent force affecting phenotypic expression. While freshwater fishes have provided a compelling example of the link between the environment and phenotypic diversity, few studies have been conducted with arid-zone fishes, particularly those that occur in geographically isolated regions where species typically inhabit intermittent and ephemeral creeks. We investigated morphological variation of a freshwater fish (the western rainbowfish, *Melanotaenia australis*) inhabiting creeks in the Pilbara region of northwest Australia to determine whether body shape variation correlated with local environmental characteristics, including water velocity, habitat complexity, predator presence, and food availability. We expected that the geographic isolation of creeks within this arid region would result in habitat-specific morphological specializations. We used landmark-based geometric morphometrics to quantify the level of morphological variability in fish captured from 14 locations within three distinct subcatchments of a major river system. Western rainbowfish exhibited a range of morphologies, with variation in body depth accounting for a significant proportion (>42%) of the total variance in shape. Sexual dimorphism was also apparent, with males displaying deeper bodies than females. While the measured local habitat characteristics explained little of the observed morphological variation, fish displayed significant morphological differentiation at the level of the subcatchment. Local adaptation may partly explain the geographic patterns of body shape variation, but fine-scale genetic studies are required to disentangle the effects of genetic differentiation from environmentally determined phenotypic plasticity in body shape. Developing a better understanding of environment–phenotype relationships in species from arid regions will provide important insights into ecological and evolutionary processes in these unique and understudied habitats.

## Introduction

Species typically exhibit considerable phenotypic variation across their geographic range (Endler 1986; Wade and Kalisz 1990; Foster and Endler 1999), where much variation can be explained by climate, habitat type, and predation pressure (Losos et al. 1998; Nagel and Schluter 1998; Schluter 2000; Langerhans and DeWitt 2004). Phenotypic variation can be attributable to numerous factors, including (1) individual genotypes producing different phenotypes in response to changing environments (i.e., phenotypic plasticity), (2) populations exhibiting fixed differences in phenotypic traits in response to selection (adaptive differentiation), and (3) processes such as genetic drift and developmental constraints (Price et al. 2003). Importantly, revealing the influence of environmental and ecological factors on phenotypic variation can provide valuable insights into evolutionary processes such as population differentiation and ecological speciation (Maynard Smith 1966; Schluter 1996; Rundle and Nosil 2005).

Freshwater fishes have provided some of the most compelling examples of the effect of environmental variation on phenotypic expression. In the guppy (*Poecilia reticulata),* for example, divergent patterns of selection associated with the risk of predation have generated populations that vary in life history traits (Reznick 1982; Reznick and Endler 1982), morphology (Alexander et al. 2006), coloration (Endler 1983), and behavior (Seghers 1974). Other selective agents may also play a role in contributing to population variation in body size and shape in guppies, including the level of canopy cover and water-flow rate (Hendry et al. 2006). Furthermore, in three-spine sticklebacks, variation in body armor (lateral plate and spine expression) has been attributed to the effects of predation, nutrient availability, and parasite abundance (Reimchen 1994; Marchinko 2009). Together, this evidence suggests that numerous and potentially interacting factors can drive patterns of phenotypic variation, thus cautioning against focusing on a single selective axis of ecological variance (e.g., high/low predation risk, benthic/limnetic habitat) when attempting to explain phenotypic variation among natural populations (Langerhans and Makowicz 2009).

Among the environmental factors influencing phenotypic variation in fishes, water flow can have an important affect on fish body shape due to the hydrodynamic effects of drag and turbulence on swimming efficiency (Enders et al. 2003; Langerhans 2008). Fishes inhabiting fast-flow habitats tend to have shallow, elongated (streamlined) bodies compared with those living in slow-flow regions, with the former becoming fusiform (spindle-shaped) through increased anterior body depth (Brinsmead and Fox 2002; Langerhans et al. 2003; Aguirre 2009; Franssen 2011; Drinan et al. 2012). Fishes with a streamlined morphology experience reduced drag and can cope with prolonged, steady swimming in moderate water flows (Schaefer et al. 1999; Wolfgang et al. 1999; Blake et al. 2005, 2009; Langerhans 2008, 2009; Blob et al. 2010). Water flow can also induce developmental shifts in morphology, as juvenile fish may develop a fusiform body shape when reared in fast flows and display a deep body form when reared in slow flows (Pakkasmaa and Piironen 2000; Paez et al. 2008). However, the reverse has also been observed, whereby fish develop deeper bodies in fast flows (Pakkasmaa and Piironen 2000; Peres-Neto and Magnan 2004; Kristjansson et al. 2012). It is likely that the complex and often unpredictable effects of water flow on body shape are in part attributable to interactions with other environmental and ecological factors.

Predation risk also has a profound effect on the phenotypic traits of many fishes, including body shape. Populations that coexist with predators typically possess deeper bodies than their low predation counterparts, which reduces their overall risk of predation relative to streamlined fishes (Andersson et al. 2006; Sass et al. 2006; Eklöv and Jonsson 2007; Chivers et al. 2008; Blob et al. 2010). In crucian carp (*Carassius carassius* L.), for example, the exposure of individuals to chemical cues from predators induces a change in body shape, such that individuals became deeper-bodied when exposed to predatory cues from the piscivorous pike *Esox lucius* (Bronmark and Miner 1992; Bronmark and Pettersson 1994). Deep-bodied fish, in turn, are able to initiate enhanced “fast-start” responses during escape from predators due to the large surface area of the body that is available to produce thrust (Law and Blake 1996; Royle et al. 2006; Domenici et al. 2008). Deep-bodied prey are also less vulnerable to gape-limited piscivores (Magnhagen and Heibo 2001; Zimmerman 2007) and require a longer handling time than slim-bodied prey (Nilsson et al. 1995).

Sexual dimorphism, which is commonly observed in freshwater fishes (Proulx and Magnan 2004; McCairns and Bernatchez 2012; Naspleda et al. 2012), is another important source of morphological variation. Divergence between the sexes can be attributable to sexual selection, for example, where certain morphologies confer mating advantages through competition or mate choice (Forsgren, 1992; Quinn and Foote 1994). In the three-spine stickleback (*Gasterosteus aculeatus*), females exhibit mating preferences for males on the basis of body morphology and size, although these preferences depend on the females’ origin; benthic females prefer large males (irrespective of shape) while limnetic females prefer slender males (Head et al. 2013). Sexual dimorphism can also arise as environmental pressures and/or constraints impact the sexes differentially. For example, female guppies can utilize high-velocity areas of streams to avoid being harassed by males (Magellan and Magurran 2006), which may result in sex-specific changes in body shape due to selection imposed by water flow on female (but not male) body shape. Accounting for factors such as sex and body size is therefore an important prerequisite for understanding the relationship between body shape variation and environmental variability.

Most studies of morphological variation in freshwater fishes have focused on species in the northern hemisphere that inhabit semi-isolated environments and display discrete ecotypes (reviewed by Skulason and Smith 1995; Robinson et al. 1996; Schluter 1996). However, the process of population divergence can be viewed as a continuum from panmixia to total reproductive isolation, and understanding the factors that constrain or promote movement along this continuum can yield significant insights into evolutionary processes (Hendry 2009). Furthermore, few studies of morphological variation have been conducted in arid or semi-arid regions, where streams are often ephemeral or intermittent and where the hydrodynamics are highly unpredictable both spatially and temporally (Dogramaci et al. 2015). Such dynamic and often disconnected conditions, at both subcatchment and basin scales, have the potential to promote the development of habitat-associated morphological specializations (Tobler et al. 2008).

Here, we investigate body shape variation in relation to habitat heterogeneity in a native Australian freshwater fish, the western rainbowfish, *Melanotaenia australis*. This species is an ideal study model for exploring the links between ecological and environmental factors and body shape. Populations occupy a wide variety of habitats in arid northwest Australia, where fish are commonly found in ephemeral pools (Morgan and Gill 2004; Beesley and Prince 2010). Males tend to be deeper-bodied and have more pointed dorsal and ventral fins than females (Allen et al. 2002), while larger males gain an advantage during mating (Young et al. 2010). Previous research on *M. australis* has revealed that variation in body shape cannot be explained by predation, but there is a tendency for morphological variation to correlate with environmental factors, such as water flow and the availability of aquatic vegetation (Young et al. 2011b). In two other species of rainbowfishes (*M. eachamensis and M. duboulayi*), variation in body shape (including fin position) is species specific and has been attributed to a number of interacting factors, including habitat, sex, and water velocity (McGuigan et al. 2003). In this study, we characterize the level of variation in adult body shape across several natural populations of *M. australis* and determine whether this variation can be attributed to specific environmental and ecological characteristics of the local habitat. In accordance with previous studies (e.g., Langerhans et al. 2003, 2007; Aguirre 2009; Harrod et al. 2010), we expected fish morphology would reflect interpopulation differences in water velocity, predation, diet, and habitat complexity. Specifically, we anticipated that deep-bodied morphs would occur in habitats with a high availability of benthic prey, high risk of predation, high habitat complexity, and slow water velocity, while streamlined morphs would be found in sites with low habitat complexity, fast water flows, and low risk of predation.

## Materials and Methods

### Study area

The study was conducted within the Fortescue River catchment in the Pilbara region of northwest Australia. The Pilbara has a semi-arid to subtropical climate, characterized by hot summers (24–40°C) and mild winters (11–26°C) (http://www.bom.gov.au). Rainfall mainly occurs in the summer, and high evaporation relative to precipitation restricts drainages to contiguous or discrete pools or reaches maintained by groundwater. Pools can vary in depth (<1.5 m to >3 m) and serve as refuges for fishes until reconnection during flooding events (Beesley and Prince 2010). Large rainfall events, primarily associated with summer cyclones, recharge catchments and contribute to sustaining pools in creeks and rivers, such that the hydrology, biogeochemistry, and the ecology of pools are intimately linked (Fellman et al. 2011). At the time of sampling, relatively little rain (<50 mm) had fallen across the catchment in the preceding 4 months. Consequently, surface water in many tributaries and along the main channel had either dried or been reduced to a series of pools maintained largely by groundwater (Fellman et al. 2011).

### Field sampling

A total of 14 sites encompassing the upper, mid, and lower subcatchments of the Fortescue River were sampled between May and November 2013. The Fortescue River is approximately 760 km in length, drains a 30,000 km^2^ catchment of the Hamersley Basin, and only flows contiguously following exceptionally large flood events (Barnett and Commander 1985). The Fortescue River is divided into upper and lower sections that are separated by the Goodiadarrie Hills (Skrzypek et al. 2013). The lower Fortescue River drains in a westerly direction from the Hamersley Ranges toward the coast, whereas east of the hills the Fortescue Marsh receives drainage from the upper catchment. The upper and lower parts of the catchment are considered hydrologically disjunct (Skrzypek et al. 2013). We have designated pools on the lower Fortescue River but still within the Hamersley Ranges as “mid-Fortescue.” Overall, four pools were sampled in the upper Fortescue, two in the mid-Fortescue and eight in the lower Fortescue. The numbers of pools sampled varied among subcatchments owing to both the number of pools that occurred in each and the local abundance of western rainbowfish.

### Habitat characterization

We collected physical environmental data (summarized in Table 1) at each site prior to capturing the fish. Five replicates of surface water velocity were measured at each pool using a water-flow probe (FP111; Global Water™, College Station, TX), which was placed 10 cm below the water's surface. At each site, the proportion of open water, fine and coarse gravel substrates, bark/wood, rocks, and aquatic vegetation were assessed to generate a habitat complexity rank (Young et al. 2011b). The rank ranged from 0 to 5, where 0 represented gravel substrates with open water (i.e., homogeneous habitat) and 5 represented dense aquatic vegetation and submerged debris (i.e., complex habitats). The depth of each pool was recorded (to the nearest 0.1 m), and pool size and shape were measured and mapped with either a tape measure or GPS, depending on size. Turbidity was measured from a 30 mL unfiltered water sample collected from each site, which was kept cool and in the dark until analyzed in the laboratory. On return to the laboratory, a turbidity meter (Hach™, Loveland, CO; model 2100A) was used to generate three replicate turbidity measures per sample.

**Table 1:** Location of sample sites, number of fish sampled, and a summary of the habitat characteristics. Complexity ranks ranged from 0 to 5, where 0 represented the lowest habitat complexity and five described the most complex sites

Site	Subcatchment	Latitude	Longitude	Fish sampled	Complexity rank	Mean surface water velocity (ms-1)	Predator presence	Proportion of surface invertebrates (%)	Invertebrate abundance	Green filamentous macroalgae (% cover)	Mean turbidity (NTU)
Outflow Creek (OC)	Lower Fortescue	−21.5762	117.0860	25	4	0.20 ± 0.03	N	88.89	9	4	1.07 ± 0.03
Deep Reach (DR)	Lower Fortescue	−21.6104	117.1074	17	1	0.00 ± 0.00	Y	NA	N/A	0	0.84 ± 0.01
Jirndawurranha Channel (JC)	Lower Fortescue	−21.5904	117.0698	27	4	0.50 ± 0.00	N	33.33	3	0	0.15 ± 0.00
Palm Pool (PP)	Lower Fortescue	−21.5702	117.0536	30	3	0.33 ± 0.11	N	0.00	10	0	0.49 ± 0.00
GB Creek (GB)	Lower Fortescue	−21.5813	117.5813	29	2	0.00 ± 0.00	Y	66.67	3	0	0.24 ± 0.01
Angular Pool (AP)	Mid-Fortescue	−22.4772	118.5631	20	0	0.02 ± 0.02	N	42.86	7	0	0.15 ± 0.00
Flat Pool (FP)	Mid-Fortescue	−22.4776	118.5567	31	3	0.14 ± 0.05	Y	33.33	3	0	0.51 ± 0.01
HD2.5	Upper Fortescue	−23.0043	119.6213	20	4	0.00 ± 0.00	N	25.00	17	20	0.39 ± 0.01
HD2	Upper Fortescue	−23.0098	119.6199	16	5	0.00 ± 0.00	N	5.51	151	10	0.76 ± 0.01
HD1.5	Upper Fortescue	−22.9897	119.6218	29	5	0.00 ± 0.00	N	22.12	35	3	0.28 ± 0.01
Kalgan (K)	Upper Fortescue	−23.1873	119.6967	4	1	0.04 ± 0.02	N	0.00	22	0	2.54 ± 0.02
Weeli Wolli 1 (WW1)	Upper Fortescue	−22.9235	119.1953	24	3	0.033 ± 0.00	N	N/A	N/A	10	0.26 ± 0.02
Weeli Wolli 2 (WW2)	Upper Fortescue	−22.9136	119.2127	20	3	0.14 ± 0.01	N	N/A	N/A	2	0.41 ± 0.01
Weeli Wolli 4 (WW4)	Upper Fortescue	−22.8827	119.2357	20	3	0.33 ± 0.02	N	N/A	N/A	0	0.27 ± 0.02

Predation pressure was determined through on-site observation and sampling, using methods developed by Young et al. (2011b) to evaluate the presence/absence of species that prey on western rainbowfish. Predators of western rainbowfish include low-risk species that prey opportunistically on rainbowfish, such as spangled perch (*Leiopotherapon unicolour*) and barred grunters (*Amniataba percoides*) and the high-risk piscivorous species such as barramundi (*Lates calcarifer*) and western sooty grunters (*Hephaestus jenkinsi*) (Young et al. 2011b). We also recorded the presence of fishing birds, such as herons. As all of the observed predators in this study were low risk, predation pressure was considered a dichotomous variable (i.e., presence or absence for each site). As specific dietary data are absent for western rainbowfish, we used dietary data from its congener the eastern rainbowfish (*Melanotaenia splendida splendida*). The diet of these two species is expected to be similar as they have similar biological and ecological roles and until recent molecular phylogenetic analysis, both were considered a part of the same species complex (McGuigan et al. 2000). The diet of the eastern rainbowfish is dominated by filamentous macroalgae (42.5%), aquatic insects (19.2%), and terrestrial invertebrates (12.3%) (Pusey et al. 2004). Each of these dietary components was therefore sampled from each habitat at the same time as fish. Surface invertebrates were measured using three 10 m sweeps using a 250 *μ*m mesh dip net. Benthic invertebrates were measured by manually disturbing (trampling) the sediments (approx. 1 m^2^ area) and sweeping a 500 *μ*m mesh D-net for 1 min before passing the sample through 2 mm and 500 *μ*m steel mesh sieves. One measure was taken from each site due to time constraints, and all invertebrates were stored in 70% ethanol. On return to the laboratory, we calculated the total number of invertebrates present, as well as the proportion of surface invertebrates comprising each sample. As the filamentous macroalgae had a highly variable distribution and abundance among pools, estimations of percentage cover were used to quantify availability.

### Fish capture and photography

Adult western rainbowfish were captured using a 10 m or 4 m seine net (both with a 6 mm mesh), depending on pool size. Juvenile rainbowfish (identified by their small size and absence of body coloration) were not used in the analysis as ontogenetic effects may contribute to variation in body shape (Paez et al. 2008). Following capture, adult fish were placed on their right side and photographed on a perspex slate with a scale bar and a mini Munsell™ Colorchecker (Grand Rapids, MI) photography standard. Photographs were taken using an Olympus™ E-PL3 (Olympus Corporation, Tokyo, Japan) camera placed on a tripod at a fixed focal length to ensure photo uniformity. Photography was performed in the shade, under natural sunlight. Fish sex was determined by placing each fish in a transparent container and examining dorsal and anal fin morphology (Pusey et al. 2004).

### Morphometric analyses of body shape

**Figure 1:**
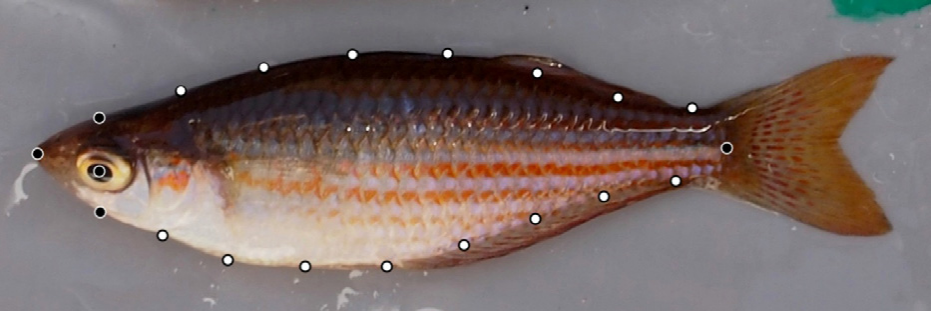
Landmark placement used in the morphological analysis of the western rainbowfish, *Melanotaenia australis*. The black markers represent the fixed landmarks while the white marks are sliding semilandmarks.

**Figure 2:**
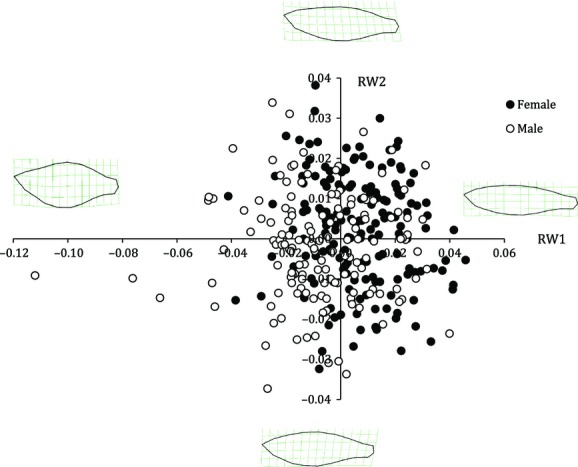
Morphological variation in RW1 and RW2 for male and female western rainbowfish captured in the Fortescue River catchment. Images illustrate the extreme morphologies represented by each axis.

Body shape was quantified using geometric morphometric analyses (Zelditch et al. 2012) and TPS software (available at http://life.bio.sunysb.edu/morph/). Twenty landmarks were assigned to the outline of each individual using TPSDIG v. 2.17 (Rohlf 2006) (Fig. 1). Five of these were fixed landmarks, which represented homologous points on the body, and were assigned to the mouth tip, center of the eye, center of the caudal peduncle edge, and on the dorsal and ventral body edges in line with the center of the eye (Fig. 1). A further 15 sliding semilandmarks were assigned to the edge of the body: seven dorsally and eight ventrally (see Fig. 1). These sliding semilandmarks were used to incorporate information about curvature in the subsequent geometric shape analysis. Together, the fixed and sliding semilandmarks produced a comprehensive shape outline that aided both the visual and analytical representation of morphological variation. The relative shape and size of fish in our sample were quantified using relative warps and centroid scores, respectively, both of which were generated using the program TPSRELW v. 1.45 (Rohlf 2005). Relative warps are principle components that quantify a change in a group of body shape attributes (e.g., streamlined to deep body shape) (Zelditch et al. 2012). Centroid size was used as a measure of body size and was calculated as the square root of the summed squared distances of each landmark from the centroid position (i.e., the average position of the 20 landmarks) (Zelditch et al. 2012). In the absence of allometry, centroid size is uncorrelated with shape; it is therefore possible to test for allometry by assessing the significance of correlations between relative warp scores and centroid size (positive or negative correlations indicate positive and negative allometry, respectively, for any given axis of shape variance). In our analysis, we focused on the first five relative warp scores (i.e., RW1–5), which explained >87% of the total variance in body shape (Fig. 2).

### Statistical modeling

A series of linear mixed-effects models combined with model selection procedures were used to evaluate the relative importance of the environmental (predictor) variables on fish morphology (relative warp scores), using the software program R (R Development Core Team 2012). We first used multivariate analysis of covariance (MANCOVA) to examine the effects of the environmental variables on total shape variation, while controlling for centroid size, which was fitted as the covariate. To visualize total variation in shape along the most important environmental axes, we used canonical variate analysis (CVA) in the software program MorphoJ (Klingenberg 2011). After evaluating total shape variation, we conducted a series of univariate tests to examine the effects of the predictor variables and their interactions on each of the relative warp scores separately. We used the lme4 package (Bates and Maechler 2009) to construct multiple models and determine the AIC (Akaike's information criterion) values of the fit of each model. The AIC values were converted into AICc values to account for the effects of sample size (Symonds and Moussalli 2011). These were used to guide model selection (models with lower AICc values were considered more parsimonious) in combination with model weights (*w*_*i*_), which can be summed for each variable (∑*w*_*i*_), to estimate their relative importance, ranging from 0 to 1 (Symonds and Moussalli 2011). Models with a change in AICc (ΔAICc) > 10 relative to the best model were excluded, while those with 6 < ΔAICc < 10 were considered unlikely and those with ΔAICc < 2 were considered equal best models (Symonds and Moussalli 2011). A weight of >0.9 and a low ΔAICc value indicates that other candidate models can be excluded (Symonds and Moussalli 2011).

We considered predictor variables that previous studies suggested were likely to affect morphology (Garamszegi 2011; Richards et al. 2011). Fixed effects included invertebrate abundance, the proportion of surface invertebrates, predator presence, habitat complexity rank, turbidity, surface water velocity, filamentous macroalgae cover, centroid size (a covariate), and sex. Site nested within subcatchment was included in every model as a random effect to account for sites that were located in the same subcatchment. A number of a priori interactions were also tested, including the interaction between size and sex (morphological differences between the sexes may depend on body size); turbidity and predation (turbidity may reduce predation risk; Snickars et al. 2004); sex and habitat complexity (due to differential habitat use by the sexes; Magellan and Magurran 2006); predation and habitat complexity (complex habitats provide refuges for prey; Walker 1997; Eklov and Svanback 2006; Sass et al. 2006); predation and sex (predators may preferentially target one of the sexes; Godin and McDonough 2003; Moyaho et al. 2004); and water flow and habitat complexity (water flow is affected by habitat; Kilsby and Walker 2012). All models were compared to a null model, which contained only the random effect. We tested the assumptions of the models by inspecting plots of the fitted models against the residual values.

## Results

### Body shape morphology

There was considerable variation in body shape among fish sampled in this study, which was predominantly attributable to differences in body depth (Table 2). Specifically, RW1, which accounted for >42% of the variance in body shape, described variation in both body depth and the length of the caudal peduncle. RW2 accounted for >19% of variation in body shape and described increased body curvature with a slightly downward-facing head to a relatively cylindrical body and an upward-facing head. In subsequent warps (RW3–RW5), changes in morphology and the proportion of variation explained were relatively minor: RW3 described reduced head length combined with increased peduncle length; RW4 described a range between downward and upward body bending; and RW5 described decreased posterior body depth. Cumulatively, RW3–RW5 explained ∼25% of the overall variation in body shape.

**Table 2:** The proportion of morphological variation explained by the first five relative warps in isolation and cumulatively. Black outlines illustrate the two relative warp morphological extremes, and orange represents consensus morphology

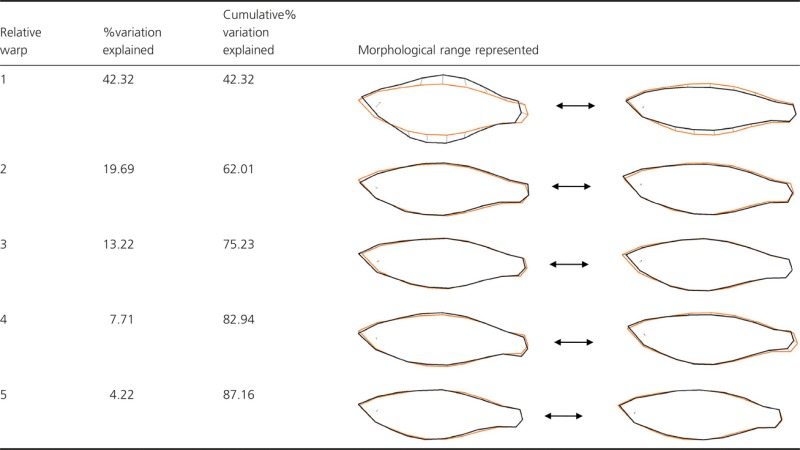

### Effect of sex and body size

**Figure 3:**
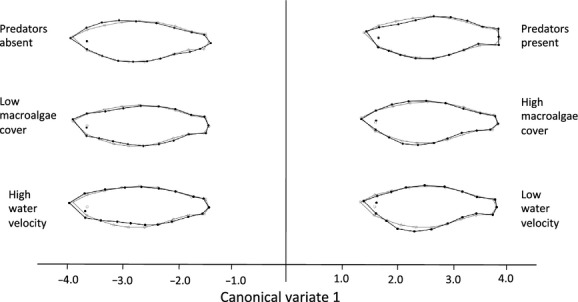
Total morphological variation associated with CV1; gray lines represent the starting shape while the black lines are the final shape. The images are for visualization purposes and represent approximately 3× the actual variation in shape. Observed CV scores ranged from −4 to 4.5 for macroalgae cover, −4.5 to 2.5 for water velocity, and −3 to 3 for predator presence.

The MANCOVA models revealed that sex was the most important determinant of total shape variation (estimate of effect of sex: −3.25e-3, SE = 6.36e-4, *t* = −5.12); models containing all the other predictor variables had AIC values >10 and were therefore considered less plausible. Extraction of the canonical variates along the major environmental axes revealed that for the first canonical variate (CV1), 38.1% of the total variation in shape was associated with water velocity and 48.0% with macroalgae cover. Positive CV1 scores were associated with slow water flows and high macroalgae cover and were linked with body deepening, while negative CV1 scores were associated with body narrowing, particularly at the anterior end (Fig. 3). Fish habitats where predators were present displayed anterior narrowing and deeper caudal peduncles (negative CV1) relative to those in habitats without predators (positive CV1) (Fig. 3).

When the sexes were analyzed separately, the top three MANCOVA models (ΔAICc < 10) describing total variation in shape contained the fixed effects of water velocity (males: estimate ± SE = 0.004 ± 0.007, *t* = 0.53; females: = 0.005 ± 0.006, *t* = 0.86), macroalgae cover (males: −0.004 ± 0.0001, *t* = −2.84; females: = −0.0003 ± 0.0001, *t* = −2.55), and predation risk (males: 0.81e-3 ± 0.003, *t* = −0.32; females: = −0.0008 ± 0.002, *t* = −0.39). For males, models containing the parameters water velocity and macroalgae were equally likely (ΔAICc < 2), while for females, water velocity was also included in the best-fitting model (ΔAICc = 0), but models containing predation risk and macroalgae also had some support and both were equally likely (ΔAICc < 2).

In the univariate tests, all of the best-fitting models (ΔAICc < 10) for RW1 included the effect of sex (∑*w*_*i*_ = 1; Table 3). Sex was also an important predictor of variation in RW2, with an overall summed weight (∑*w*_*i*_) of 0.62. The predictor “sex” also appeared in the top models for RW4 and RW5; however, these models were poor candidates due to low weights (RW4: *w*_*i*_ = 0.17; RW5: *w*_*i*_ = 0.02) and relatively large AICcs (ΔAICc RW4: 3.32; RW5: 8.32). In fact, models for RW2, RW4, and RW5 did not significantly differ (ΔAICc < 2) from the null model, which contained only the random effects, suggesting that none of our predictor variables accounted for variation in these body shape attributes. The RW1 scores revealed that males displayed deep bodies, short caudal peduncles, and a curved body, while females tended to have slimmer, bullet-shaped bodies with upward-facing heads. Centroid size was included in all of the top RW1 and RW3 models (ΔAICc < 10), indicating allometry in body depth, head, and peduncle length.

**Table 3:** Linear mixed models with a ΔAICc < 10 testing the effects of environmental and ecological variables on body shape variation. Each model contained the random site nested within subcatchment term. The Akaike weight (*w*_i_) indicates the level of confidence (0–1) that the model selected is the best model, or when summed, the relative importance of the predictor variables. The percentage of variance associated with the random effects is shown, along with the residual variance

Dependent variable	Model terms	Nested variance (%)	Subcatchment variance (%)	Residual variance (%)	AICc	ΔAICc	*w*_*i*_	Fixed effect estimate
RW1	Site (subcatchment) + sex + centroid size	19.36	50.63	30.02	−1777.96	0.00	0.85	Sex: −1.10e-02 ± 1.40e-03
								Centroid: −2.60e-05 ± 3.71e-06
	Site (subcatchment) + sex + surface velocity + centroid size	10.96	61.83	27.22	−1774.47	3.48	0.15	Centroid: −2.62e-05 ± 3.70e-06
								Sex: −1.09e-02 ± 1.40e-03
								Surface velocity: 4.10e-02 ± 1.66e-02
	Site (subcatchment) + sex + centroid size + predation	20.98	49.67	29.36	−1767.99	9.96	0.01	Sex: −1.10e-02 ± 1.40e-03
								Centroid: −2.60e-05 ± 3.71e-02
								Predation: −8.47e-05 ± 7.89e-03
RW2	Site (subcatchment) + sex	15.05	12.62	72.34	−1827.34	0.00	0.61	Sex: −5.24e-03 ± 1.37e-03
	Site (subcatchment)	13.92	11.13	74.95	−1826.36	0.98	0.38	–
	Site (subcatchment) + sex + surface velocity	17.17	11.13	71.71	−1818.47	8.87	0.01	Sex: −5.23e-03 ± 1.37e-03
								Surface velocity: 4.45e-03 ± 1.28e-02
	Site (subcatchment) + surface velocity	15.88	9.42	74.70	−1817.56	9.78	0.01	Surface velocity: 5.97e-03 ± 1.25e-02
RW3	Site (subcatchment) + centroid size	23.65	7.95	68.40	−2033.34	0.00	0.90	Centroid: 2.55e-05 ± 2.52e-06
	Site (subcatchment) + centroid size + surface velocity	19.50	2.18	78.320	−2028.91	4.43	0.10	Centroid: 2.57e-03 ± 2.51e-06
								Surface velocity: -2.50e-02 ± 8.92e-03
RW4	Site (subcatchment)	14.34	0.00	85.66	−2091.72	0.00	0.82	–
	Site (subcatchment) + sex	14.43	0.00	85.57	−2088.58	3.32	0.17	Sex: −3.02e-03 ± 9.01e-04
RW5	Site (subcatchment)	15.36	20.90	63.74	−2344.90	0.00	0.91	–
	Site (subcatchment) + centroid size	18.72	20.96	60.31	−2338.99	5.91	0.05	Centroid: 7.21e-06 ± 1.58e-06
	Site (subcatchment) + surface velocity	11.05	22.22	66.72	−2337.78	7.12	0.03	Surface velocity: 1.01e-02 ± 4.92e-03
	Site (subcatchment) + sex	17.10	19.53	63.37	−2336.67	8.32	0.02	Sex: 1.57e-03 ± 5.97e-04

### Environmental predictors

None of the measured environmental fixed effects (predator presence, habitat complexity rank, turbidity, surface water velocity, filamentous macroalgae cover, sex) was included in the top model set for any of the relative warp scores. There was a common reoccurrence of surface water velocity in the suboptimal models for RW1, RW2, RW3, and RW5; however, these models were poor candidates owing to their AICc values (ΔAICc 3.48–9.78) and low weights (0.01–0.15) (Table 3). Predation was present in one of the RW1 models but had low weight (0.01) and an AICc value that was considerably higher (indicating a poorer fit) than the top model (ΔAICc = 9.96). Conducting partial correlations (controlling for centroid size) on the mean relative warp scores with the site means for the environmental parameters revealed a significant negative correlation between both RW2 and RW5 and the mean percentage cover of macroalgae (RW2: *r*_13_-0.68, *P* = 0.008; RW5: *r*_13_ = −0.69, *P* = 0.006), and a negative correlation between RW3 and mean water velocity (*r*_13_ = −0.53, *P* = 0.049). This result suggests that increasing macroalgae cover is associated with a downward-facing head and increased body curvature while increased water velocity is linked with changes in head and caudal peduncle length.

### Geographical effects of subcatchment and site

Subcatchment and site were included in all optimal models, and the relative importance of each varied depending on the relative warp (Table 3). Subcatchment explained a higher proportion of the random variation than site in the optimal models for RW1 (50.6% compared to 19.4% for site) and RW5 (20.9% compared to 15.4% for site). Subcatchment variance was lower (0.0–12.6%) than (nested) site variance in all other relative warps (14.3–23.6%) in the optimal models.

**Figure 4:**
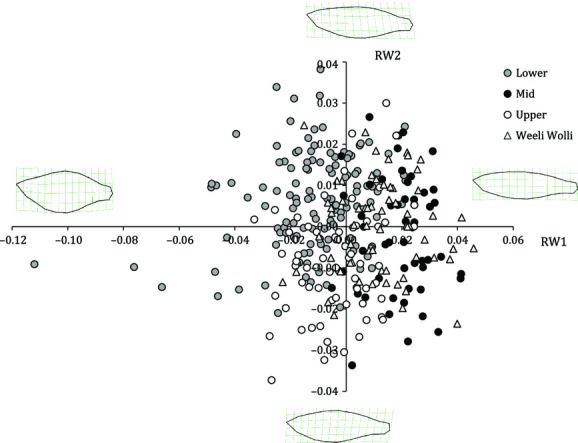
Morphological variation in RW1 and RW2 characterized by three subcatchments of the Fortescue catchment (upper, mid, and lower; white, black, and gray symbols). Weeli Wolli Creek sites (with artificially modified flow) are shown separately (triangle symbols) as they vary from the typical upper subcatchment pattern. Images illustrate the extreme morphologies represented by each axis.

**Figure 5:**
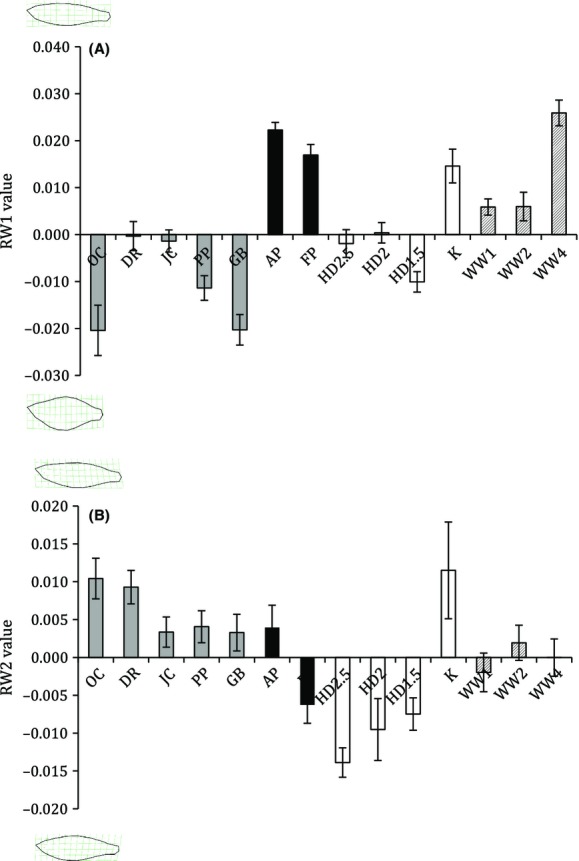
Among-population morphological variation in RW1 (A) and RW2 (B). Images illustrate the extreme morphologies represented by each axis. Shading indicates the subcatchment classification for each population (upper: white, middle: black, lower: gray) with Weeli Wolli Creek (WW) sites (artificially modified flow; hatched) shown separately (see Table 1 for individual site codes).

RW1 and RW2 formed three distinct morphological groups when plotted according to subcatchment, particularly when considering variation along the RW1 axis (Fig. 4). The mid-Fortescue populations had slim bodies while the lower Fortescue populations tended to have deep, cylindrical bodies with shorter caudal peduncles and upward-facing heads. The upper Fortescue populations tended to have downward-facing heads and increased body curvature relative to the lower and mid subcatchment sites. However, not all populations fell into this distinct pattern. In particular, fish from the Weeli Wolli Creek sites (which have received continuous artificial discharge from mine de-watering since 2007; Dogramaci et al. 2015) tended to be morphologically more similar to those in the mid-Fortescue populations (i.e., slim-bodied) than those from other sites in the upper Fortescue (Fig. 5).

## Discussion

We found that western rainbowfish exhibited considerable variation in body shape and marked sexual dimorphism, with males displaying deeper bodies and shorter caudal peduncles than females. Geographic patterns of morphological differentiation were largely consistent with the species’ distribution among discrete subcatchments. Nevertheless, some populations did not conform to this geographic pattern and levels of morphological similarity among subcatchments were not consistent with the hierarchical stream order (i.e., pool – creek – subcatchment – catchment). The environmental factors measured in this study, including water velocity, habitat complexity, the presence of predators, and food availability, played only a minor role in explaining overall variation in body shape. Consequently, our findings suggest that, at the population level, variation in habitat characteristics may not be the main driver of phenotypic diversity; instead, the morphological variability of the western rainbowfish may reflect underlying patterns of genetic differentiation and sex-specific selective pressures.

Our findings contrast with previous studies that have documented a strong effect of water velocity (Brinsmead and Fox 2002; Langerhans et al. 2003; Aguirre 2009; Franssen 2011; Drinan et al. 2012), predation pressure (Basolo and Wagner 2004; Langerhans and DeWitt 2004; Langerhans et al. 2007), and habitat type and diet (Hjelm et al. 2001; Berner et al. 2008; Svanback et al. 2008; Aguirre 2009) on the morphology of wild-caught fishes. A possible explanation for this point of difference is that we did not measure the morphology of the fins, which are important determinants of thrust, drag, and maneuverability while swimming (Plaut 2000; Drucker and Lauder 2001; Nauen and Lauder 2002). For example, caudal fins provide thrust and counteract drag while swimming. Thus, the height of the caudal fin is often increased in fish that are exposed to fast-flowing water (Pakkasmaa and Piironen 2000; Brinsmead and Fox 2002; Imre et al. 2002). We did not measure the fins because extending and positioning the fins for photography is difficult on live, nonanaesthetized fish.

Previous studies have revealed that freshwater fishes commonly exhibit sexual dimorphism for both body size and shape (e.g., Quinn and Foote 1994; Caldecutt et al. 2001; McGuigan et al. 2003; Hendry et al. 2006). Our analysis of body shape supports prior observations that male rainbowfish tend to be deeper-bodied than females (Allen et al. 2002). Sexual selection *via* female choice and male–male competition commonly explains sexual dimorphism, and both of these processes operate in *M. australis* (Young et al. 2010). Although female western rainbowfish prefer large males, it would be interesting to investigate whether there is also sexual selection on body shape and whether this varies among populations (Head et al. 2013). It would also be worthwhile investigating the functional significance of the observed sexual dimorphism; a previous study with other species of rainbowfishes found that variation in body shape was not attributed to hydrological habitat, but the sexes differed in critical swim speeds (McGuigan et al. 2003). These findings suggest that environmental factors such as water flow may lead to differential habitat use by the sexes and sexual segregation.

Sexual dimorphism may also arise due to natural selection operating differentially on the sexes. The sexes are often exposed to different levels of predation risk, for example, because bright colors that attract females also increase the males’ conspicuousness to visual predators (Godin and McDonough 2003; Moyaho et al. 2004). Male rainbowfish tend to be more brightly colored than females (Young et al. 2011b) and may therefore compensate for their increased risk of predation by developing deeper bodies that provide protection from gape-limited predators (Nilsson et al. 1995; Domenici et al. 2008). However, in this study, predator presence was associated with an overall narrowing of the anterior of the body while predator absence was associated with body deepening. This finding might be an adaptation for fast-start escape responses from predators because a narrow head should reduce drag while a deep caudal peduncle should increase thrust (Langerhans and DeWitt 2004). We also found some evidence for an effect of macroalgae cover on increased body deepening and a downward-facing head, which could be advantageous for prey capture efficiency and navigation in complex habitats (Blake et al. 2005; Eklov and Svanback 2006; Sass et al. 2006; Svanback and Eklov 2006). In short, differences between the sexes in morphological traits may reflect a complex interplay between natural and sexual selection that ultimately favor different body morphologies in males and females.

Most of the variation in our morphological data was explained by geographic (subcatchment) effects, which might be explained by underlying genetic differentiation or the distinctive habitat characteristics of each region, or an interaction between the two. A recent study of the western rainbowfish found that morphological variation across the species’ range is consistent with a hierarchical pattern of genetic divergence (Young et al. 2011a), lending support to the notion that, on a regional scale (among drainages), local adaptation is the main process driving phenotypic differentiation and may have contributed to the evolution of body shape polymorphism in this species (Young et al. 2011a). Wet season dispersal is considered to be an important determinant of genetic structuring in *M. australis*, particularly over small spatial scales such as within creek lines (Phillips et al. 2009). In the present study, sites that are located within the same creek lines are in relatively close proximity (within 40 km) and likely to become connected during summer rainfall (Fellman et al. 2011). In contrast, subcatchments are isolated by large distances (∼180 km), with the upper subcatchment being separated from the mid and lower subcatchments by the Goodiadarrie Hills (Barnett and Commander 1985; Skrzypek et al. 2013). Nonetheless, recent studies of the biogeography of the Pilbara, an area of some 500,000 km^2^, have revealed that patterns of genetic diversification are often not consistent with biogeographical region, particularly at a fine spatial scale (Pepper et al. 2013). Indeed, there is increasing evidence that the Hamersley Ranges of the Pilbara (encompassing the upper subcatchment of our study is characterized by high levels of endemism and “cryptic diversity” of both animal and plant species (Cracraft 1991; Unmack 2001)).

Aspects of our study have highlighted some of the challenges of conducting research on fishes inhabiting arid and remote regions; sample sites are often sporadic and separated by hundreds of kilometers, which means that sample size is necessarily limited. In the current study, 14 sample sites were split across three distinct geographic subcatchments. We considered the geographical features of the landscape using subcatchment as a random effect; however, the effect of region may have masked any responses to the environmental parameters, particularly because not all habitat types were represented in each region. This may have limited our ability to detect any environmental effects or their interactions. Hydrological conditions in the study region are also typically highly variable among years, leading to unpredictable environmental conditions and strong temporal effects on pool connectivity. The observed morphological variation we observed may therefore reflect past ecological conditions, that is, hydrological events occurring during the fish's development, which would encompass periods of several months, rather than necessarily the conditions at the time of sampling. Indeed, a lack of morphological differentiation in pumpkinseed fish (*Lepomis gibbosus*) has been attributed to the strong seasonal variation in water flows that occurs in the Mediterranean, suggesting that dynamic environments may confound morphological responses (Naspleda et al. 2012). Furthermore, flow at one of the creeks in this study (Weeli Wolli Creek; encompassing three sample sites) has been modified for ∼7 years due to mining activities in the area. This previously ephemeral creek now has continuous surface flows for ∼24 km due to the discharge of groundwater (Dogramaci et al. 2015). Interestingly, fish from these sites tended to possess slender bodies relative to other populations within their (upper) subcatchment, perhaps due to the occurrence of relatively fast-flowing water (>0.4 m^−1^s) at this site. This finding is consistent with models of fish swimming biomechanics; fish exposed to fast-flowing water are expected to develop fusiform body shapes for optimal steady swimming performance while those in low flows should develop deeper bodies that maximize thrust and stability for “burst” swimming (Langerhans 2008). Although previous fish studies have reported morphological responses to anthropogenic modifications such as impoundment (Haas et al. 2010; Franssen 2011; Franssen et al. 2012, 2013), further sampling of streams receiving high rates of discharge is required to determine whether the apparent morphological response observed here can be generalized.

In summary, we found limited evidence that body shape variation in *M. australis* corresponded with strong habitat differentiation or in response to differences in key environmental factors such as water flow. However, patterns of morphological differentiation were largely consistent within three geographically distinct subcatchments, suggesting that these regions may present distinct and isolated habitats that may promote differentiated patterns of morphology. Nonetheless, fine-scale genetic studies are required to determine whether patterns of genetic structuring are consistent with the geographic features of the Pilbara landscape and its hydrological connectivity. We found some evidence that fish have altered their morphology in a creek affected by mine dewatering; however, further study is required before we can establish whether this provides an example of morphological responses to anthropogenic habitat alteration (Franssen 2011; Franssen et al. 2012, 2013).

## Data accessibility

Data will be made publicly available on the general-purpose repository Dryad (https://datadryad.org).
